# The Autoimmune Disease Database: a dynamically compiled literature-derived database

**DOI:** 10.1186/1471-2105-7-325

**Published:** 2006-06-27

**Authors:** Thomas Karopka, Juliane Fluck, Heinz-Theodor Mevissen, Änne Glass

**Affiliations:** 1 Institute for Medical Informatics and Biometry, University of Rostock, Rembrandt-Str. 16/17, 18055 Rostock, Germany; 2 Fraunhofer Institute for Algorithms and Scientific Computing (SCAI), Department of Bioinformatics, Schloss Birlinghoven, D-53754 Sankt Augustin, Germany

## Abstract

**Background:**

Autoimmune diseases are disorders caused by an immune response directed against the body's own organs, tissues and cells. In practice more than 80 clinically distinct diseases, among them systemic lupus erythematosus and rheumatoid arthritis, are classified as autoimmune diseases. Although their etiology is unclear these diseases share certain similarities at the molecular level i.e. susceptibility regions on the chromosomes or the involvement of common genes. To gain an overview of these related diseases it is not feasible to do a literary review but it requires methods of automated analyses of the more than 500,000 Medline documents related to autoimmune disorders.

**Results:**

In this paper we present the first version of the Autoimmune Disease Database which to our knowledge is the first comprehensive literature-based database covering all known or suspected autoimmune diseases. This dynamically compiled database allows researchers to link autoimmune diseases to the candidate genes or proteins through the use of named entity recognition which identifies genes/proteins in the corresponding Medline abstracts. The Autoimmune Disease Database covers 103 autoimmune disease concepts. This list was expanded to include synonyms and spelling variants yielding a list of over 1,200 disease names. The current version of the database provides links to 541,690 abstracts and over 5,000 unique genes/proteins.

**Conclusion:**

The Autoimmune Disease Database provides the researcher with a tool to navigate potential gene-disease relationships in Medline abstracts in the context of autoimmune diseases.

## Background

Autoimmune diseases are commonly considered complex immune disorders. While many autoimmune diseases are rare, collectively these diseases afflict millions of patients. According to [1] 5–8% of the US population suffers from this group of chronic, debilitating diseases. Despite their clinical diversity, they have one similarity, namely the dysfunction of the immune system. It is suspected that genetic defects play a role in the etiology of these diseases. Modern high throughput technologies, like mRNA microarrays, have enabled researchers to investigate diseases at a genome-wide level. In contrast to classical inherited genetic diseases, like sickle cell anemia, autoimmune diseases are not caused by the defect of a single gene, but by the dysfunction of the complex interaction of a group of genes. Although no autoimmune disease has been completely analysed, there has been tremendous success in recent years in identifying major players in the development of autoimmune diseases. In [2] there are over 50 publications that list gene variants that are associated with a certain autoimmune disease. Interestingly, a lot of these genes are located in the same regions on the chromosomes, the so called susceptibility regions. This has led to a "common cause hypothesis" of autoimmune disorders. Several organisations and institutes have established programs to investigate this common cause hypothesis, among them the American Autoimmune Related Diseases Association (AARDA) [3], the Autoimmune Diseases Research Center at the Johns Hopkins Medical Institutes [4], the Autoimmune Diseases Research Foundation (ADRF) [5] and the Multiple Autoimmune Disease Genetics Consortium (MADG) [6]. However, defects of one or more of these genes do not cause an autoimmune disease, but only predispose a person for an autoimmune disease.

The factors that trigger an autoimmune disease are still unknown. Studies with monogenetic twins have revealed that genetic influences only account for 25–40% of the disease risk making gene environment interactions or environmental influences the predominant factors. The environmental influences are very diverse rendering research in this area extremely difficult. These influences may be toxic substances like mercury in one case and ultraviolet light or even certain nutrients in another. Moreover, several bacteria, viruses or hormones are among the suspected triggers of autoimmune disorders.

In the post genomic era researchers are confronted with the phenomenon that while the amount of accessible data is growing exponentially, it is becoming harder and harder to find the appropriate information. The number of biomedical databases listed in the Nucleic Acids Research 2005 Database Issue [7] has increased by 171 to 719. However, while information for entities like genes or proteins can be found in databases like GenBank or InterPro, information about relations between entities is still scarce. The main information source is still free text. In the recent years, a lot of research has been done in the field of information extraction and text-mining. State-of-the-art systems are now able to recognise gene or protein names with a precision between 80 – 95% and a corresponding recall between 80 and 90% depending on the organism [8]. In light of such capabilities it has become viable to use these techniques in the compilation of databases. Several projects are already using text-mining to support the human experts that curate databases. For instance the curation of the protein-protein interaction database BIND [9] is supported by a program called PreBIND [10] and the Molecular Interaction Database (MINT) [11] is supported by the MINT Assistant [12]. In these examples software is utilised for information extraction by filtering relevant documents and thus lowering the amount of work for human experts. To cover the broad range of all autoimmune diseases, we still deal with over 500,000 Medline abstracts, a number much to high for any human expert to browse. We therefore opted for the compiling of the database in a fully dynamic way. Using text-mining allows the dynamic compilation of a database enabling researchers to gain an overview of this extensive field.

In this paper we present a web-based database designed to support researchers in the area of autoimmune diseases. In the first section we will describe the design of the database itself as well as the techniques used to compile the database. Furthermore, the generation of comprehensive synonym lists for the disease areas and the ProMiner system [13] used for the recognition of protein and gene names will be described. In the utility section we will explain the content, different views and query capabilities of our database. We also provide an evaluation of the database content. In the discussion section we will briefly review similar work in this area and address the unique features of our system in particular.

## Construction and content

The Autoimmune Disease Database (AIDB) [14] is a relational, integrated database that was dynamically compiled using dictionary based approaches for named entity recognition of disease terms and protein and gene names. For the autoimmune disease terms the Medical Subject Headings (MeSH) [15] were used and additionally a dictionary based on the Unified Medical Language System (UMLS) [16] was developed. For the search every term in the list was sent as query to the PubMed database [17]. The recognition of gene and protein names was based on a named entity recognition system (ProMiner) which uses a dictionary generated out of the Entrez Gene and Swiss-Prot entries. Table 1 shows some statistics of the database content.

The basic underlying concept behind the AIDB is that of co-occurrences combined with statistical ranking between disease terms and protein/gene names. Whereas we have to accept a certain error rate due to the fact that the pure mentioning of a disease in combination with recognised gene and protein names does not imply a direct relationship, this simple method allows us to gain a quick overview over a huge amount of abstracts and a high retrieval rate concerning possible relationships. Hypothetic relations can be manually verified through the link to the relevant text sources.

The Web-Presentation is designed using PHP and JavaScript. For the storage of the data MySQL 4.0.13 is used.

### Compilation of an autoimmune disease dictionary

The list of autoimmune diseases used in this database was compiled from several sources, among them lists from the American Autoimmune Related Diseases Association (AARDA) [3], Johns Hopkins Autoimmune Diseases Research Center [4] and MeSH. Some experts might disagree whether one or the other of the listed diseases can be really considered as an autoimmune disease. But this does not harm our analysis. On the contrary, it could be interesting to include other diseases like asthma or allergic diseases which also share some similarities with autoimmune diseases as pointed out in [18]. In the current version of the AIDB we would like to concentrate on the core list as defined above.

One problem that has to be tackled when applying text mining techniques is synonymy. In the case of diseases, there often exist different names for the same disease. Looking only at certain names therefore gives an incomplete picture. We solve this problem by using the UMLS. The UMLS is an umbrella system that unifies over 60 distinct clinical terminologies. The basic organisational unit in the UMLS is a concept. Each concept has a concept unique identifier (CUI). Other organisational units are the string unique identifier (SUI) and the language unique identifier (LUI) which are used to handle string variants and language variants respectively. Each autoimmune disease is represented by a concept (and therefore by a CUI). All known synonyms are linked to this concept. An example is given in table 2. This table lists all synonyms for the concept "Takayasu's Arteritis". This concept has 29 synonyms or orthographic variants. A complete list of the autoimmune disease concepts in the AIDB can be found on the "Browse Disease" page [19]. The current version contains 1,220 synonyms and orthographic variants for 103 concepts.

The usage of concepts as described above results in a higher retrieval rate, in comparison to MeSH, the National Library of Medicine's controlled vocabulary for indexing Medline. There are 79 MeSH terms for autoimmune diseases but 103 disease concepts extracted out of the UMLS. But because the indexing is done by human experts the quality of the assigned MeSH terms is quite high. Even if the MeSH indexing can not be considered complete and retrieve fewer matches, the usage of the MeSH terms results in a higher precision.

Therefore we integrated two different search methods in our system: the search of the whole disease synonym list to increase the retrieval of matches and the use of MeSH terms to have a higher certainty in respect to the recognised disease terms. The database contains a table for disease concept-PMID links and a table for MeSH term-PMID links. These tables are compiled using a Java Program and the Entrez programming utilities [20]. In the case of UMLS concepts, the program sends a query for every term in the list to the PubMed database using the "[tiab]" qualifier to restrict searches to "Title and Abstract". In the case of MeSH terms, the list of MeSH terms is used combined with a "[mesh]" qualifier resulting in a table of all PubMed abstracts indexed with an autoimmune disease as MeSH term.

For the recognition of gene and protein names, which raises many more recognition problems, we used an already established software (ProMiner) [13] which is briefly described in the next section.

### Dictionary-based named entity recognition of gene and protein names in the ProMiner system

The ProMiner system consists of three different modules. The first module covers the generation and curation of a gene/protein name dictionary, which associates each biological entity with all known synonyms. The synonyms are extracted out of the Entrez Gene database [21] and the Swiss-Prot database [22]. As the name and synonym fields in these databases often contain physical descriptions (e.g. *cDNA clone*, *RNA*, *5'end*), family names (e.g. *membrane protein*) or other annotation remarks, the dictionary is cleaned up by an automated process. Each synonym is classified into one of several classes, which are associated with specific parameter settings in the subsequent search queries.

The second part of the system consists of an approximate search procedure which is geared towards high recall and accepts different parameter settings for each of the synonym classes (e.g. search case sensitive or insensitive, with or without permutations). This procedure is applied to detect all potential name occurrences on the basis of the constructed dictionary. Each synonym is treated as a string of letters which can be split into several tokens. These tokens generally correspond to words or numbers. For instance, the string 'TGF-beta receptor type 3 precursor' would be split into seven tokens: 'TGF', '-', 'beta',' receptor', 'type', '3' 'precursor'. The detection problem is addressed on the level of such tokens. Tokens are equivalent if their strings match exactly. Depending on the parameter the case of the strings has to match as well. Furthermore, the set of all tokens is categorised according to token classes which vary in significance for occurrence detection. For the example above, the tokens "TGF", "receptor", "3" are of higher relevance for a match than the tokens "-", "type" or "precursor".

The search procedure works by browsing over the abstract, processing one token at a time and keeping a set of candidate solutions for the respective position. Each candidate solution is associated with two scoring measures. One scoring measure, the boundary score, controls the end of the extension of a candidate match and is increased on a token mismatch. If this score rises above a defined threshold, i.e. if a certain number of mismatches have occurred, the candidate is pruned from the candidate set and checked for reporting. Then, the second score measure, the acceptance score, determines whether the candidate is reported as a match. The acceptance score is a linear combination of token class specific match- and mismatch terms. A match term is defined as the percentage of matched tokens of the respective token class. A mismatch term counts for each token class the number of tokens additionally found in the text and, thus, mismatched in the candidate synonym. With appropriate weighting, the acceptance score allows to accept variations of synonyms and, at the same time, disregard false substring matches. In such a way the approximate search strategy in ProMiner allows for the recognition of different spelling variants of dictionary entries in text.

In a last step, filters are applied to increase specificity of the search results. The disambiguation filter attempts to resolve ambiguous matches. This is important for the resolution of overlapping matches (e.g. the protein name 'TGF' should not match 'TGF receptor') but also to accept only unique matches in the case of ambiguous terms. A match is called unique if the match in the text could be associated only to one Entrez Gene entry. If two or more different gene entries share a synonym (e.g. LPS is used as synonym for the Entrez Gene entries 3664 and 7452) the system only accepts the match for the gene entry if it finds another synonym for the same gene entry in the text (e.g it would additionally find IRF6 for Entrez Gene entry 3664). A synonym might also be ambiguous because it is an acronym used in different contexts (e.g. LPS is mostly used as an acronym for lipopolysaccharide). Here names from acronym dictionaries are additionally detected in the text to resolve these ambiguities.

The ProMiner system was recently tested in the BioCreAtIvE assessment for the detection of gene and protein names [23] for the organisms mouse, fly and yeast. The ProMiner systems achieved the best performance in F-score for mouse (0.79) and fly (0.81), and for yeast the second best (0.9) (cf. table 3). For human we created our own benchmark set with 250 annotated abstracts and reach similar performance (F-score = 0.84, cf. table 3). In the BioCreAtIvE assessment we also tested to accept matches for non-unique gene and protein names. Here a recognised name in the text could be matched to up to three different gene entries if the recognised synonym is associated with these entries. It was shown that we get higher recall but at the same time lose precision and also overall performance in F-score (cf. table 3, fly, accept matches of synonyms associated with up to 3 different Entrez Gene entries). For AIDB we therefore chose to accept only unique matches.

## Utility

The aim of the AIDB is to provide the researcher with a quick overview of potential links between genes/proteins and autoimmune diseases. The AIDB can be searched through a web interface at [14]. There are two main starting points for searching the database depending on the question the user tries to answer. The user can either browse the list of disease concepts or MeSH terms, or use the search box in the top left corner to search for disease terms or genes of interest. These two scenarios are described in detail below.

### Disease queries

**Figure 1:**
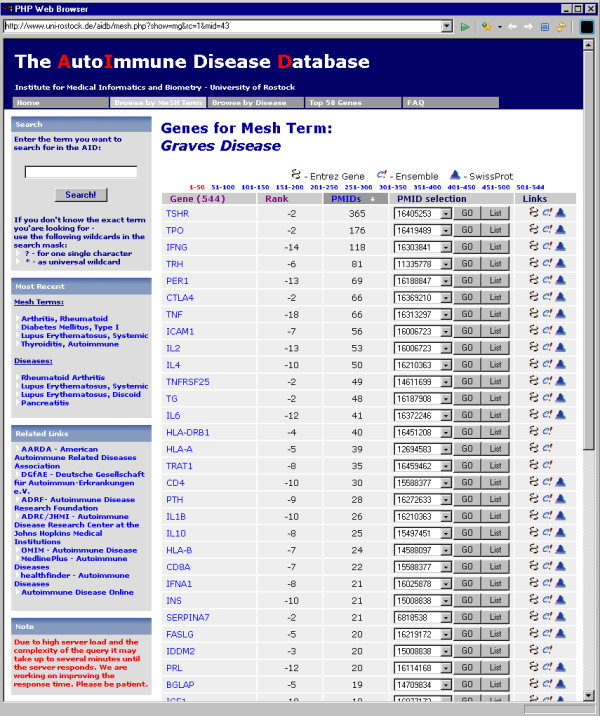
**Disease query for the MeSH term Grave's disease**. Screen shot for the MeSH term "Graves' disease". The first column (Gene) contains the gene name. Moving with the mouse over the gene name shows the synonyms in a tool-tip. Clicking on the gene name initiates a search for this gene in the concept space. The second column contains the RANK coefficient, the third column the number of corresponding PMIDs and the forth column contains a pull-down menu containing the PMIDs. Like in the Concept view it is possible to link to the PubMed abstracts via the "Go" or the "LIST" button showing the abstract corresponding to the actual PMID or all abstracts respectively. The last column contains links to Entrez Gene, Ensembl and Swiss-Prot for further information about the current gene or protein.

Disease queries are either initiated on the "Browse by disease" page or on the "Browse by MeSH term" page. The difference lies in the construction of the underlying association tables. For the disease link we have searched the title and abstract of all Medline abstracts for the disease terms using the PubMed search interface. For the gene-MeSH term links we searched the MeSH entries for the abstracts using the PubMed search interface. The reason for this distinction is that not all autoimmune diseases are listed as MeSH terms. Furthermore not all abstracts are indexed. These abstracts are covered in the disease view. The whole corpus of Medline abstracts that mention an autoimmune disease or are indexed with an autoimmune disease as MeSH term consists of 541,690 entries. 52% of these abstracts have both an autoimmune disease as MeSH and a concept in the free text. But 23% only have a disease name in the abstract, and 25% only have a MeSH term. As the views are very similar, we only show the MeSH view here. Figure 1 shows a screen shot for the search of all genes found for "Graves' disease" sorted descending by number of PMIDs. The user has now the option to either directly go to the reference for each gene shown in the drop down list in the third column by pressing the "Go" button, or to browse the whole list of references by pressing the "List" button. When pressing the "List" button the complete list of references is presented as sorted by publication date. Disease terms and recognised gene or protein names are highlighted in the abstracts to support a quick understanding of the described gene-disease association. In the last column three links are provided: Entrez Gene, Ensembl and Swiss-Prot. With these links, the user can obtain a detailed overview of the specific gene or protein of interest.

**Figure 2:**
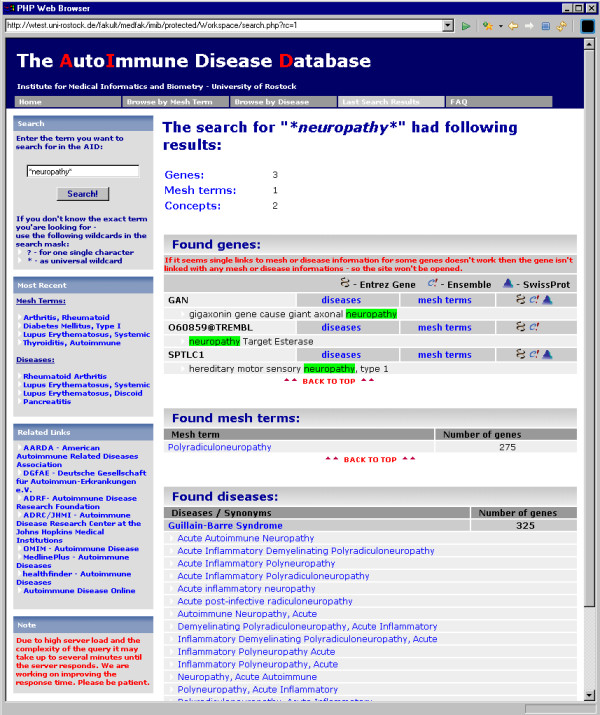
Result for the search of the string "*neuropathy*". Screen shot for the search of the string "*neuropathy*". In the first section all genes are listed that contain the search string. The search term is highlighted in green. The following two sections show the matching MeSH terms and disease concepts respectively.

Furthermore the search box in the upper left corner can be used to search for disease concepts, MeSH terms or genes. Wildcards may be used if the user does not know the exact term. Figure 2 shows the result for the query "*neuropathy*".

### Gene queries

**Figure 3:**
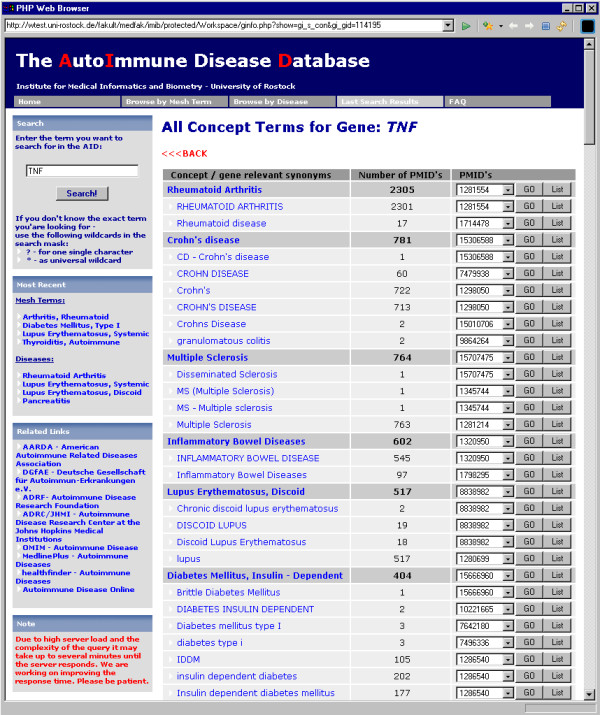
**Gene query for the Gene TNF**. Screen shot for a gene query for the gene TNF. All autoimmune concepts and disease names that co-occur with TNF or one of its synonyms are listed on the page. Each line corresponds to a concept or disease. The second column shows the number of corresponding PMIDs indicating how frequent the terms are co-mentioned. The last column contains all PMIDs in a drop down list. Pressing the "Go" button links to the original abstract in the PubMed database of the PMID currently shown in the drop down list. Pressing the "List" button opens a new window containing all abstracts contained in the drop down list.

For a gene query the user types a gene name or gene symbol into the search box. The database maintains a list of gene symbols or official names as standardised by the HUGO Gene Nomenclature Committee (HGNC) [24]. There is also a list of synonyms for each gene or protein. If we do not have an official name or a synonym, the Entrez Gene identifier is used. Additionally we provide the names as they are used in the PubMed abstracts. If the user moves the mouse over a gene symbol, a list of alternative symbols and names is shown in a tool-tip box. After the user initiates the search the gene name and synonym tables are searched and the user is provided with a list of matches. The user is then provided with an option to browse the list of associated disease concepts or MeSH terms by pressing on the "disease" or "MeSH term" button respectively. Figure 3 shows a list of all autoimmune diseases that co-occur in a PubMed abstract for the gene "TNF" after pressing the "disease" button. If no gene-disease association is recorded for this gene in the AIDB this view will not show up.

### Measuring the relevance of gene-disease links

Given the high number of gene-disease links for most of the disease concepts it is highly desirable to have some kind of measure to evaluate the relevance of the different genes to a particular disease. We have implemented three methods (GENE, PMID, RANK) to sort the list of genes. The first method allows to sort the gene names alphabetically (GENE). The second method is based on the frequency of the genes in the context of the considered disease. Genes are listed according to the number of co-occurrences with a disease in PubMed references (PMID). The third method (RANK) calculates a relevance score as described below. This method was introduced in [25] to measure the relevance of gene-to-disease links. In the following we explain how we applied this method in the context of autoimmune diseases.

In brief, the method allows measuring the strength of the relationship between a protein and a disease by analysing how much the observed number of "protein documents" deviates from the expected number if the draw had been random. To illustrate the method we give an example for IL4 in the context of insulin-dependent diabetes mellitus (IDDM).

First we define the Set S which consists of all Medline abstracts that contain a gene or protein name that was recognised by the ProMiner software. N is the number of all documents in this set and n_IL4 _is the number of abstracts where IL4 is mentioned. With these two figures we can calculate the probability (1) of the occurrence of the term IL4 in a document of this set.

pIL4=nIL4N     (1)
 MathType@MTEF@5@5@+=feaafiart1ev1aaatCvAUfKttLearuWrP9MDH5MBPbIqV92AaeXatLxBI9gBaebbnrfifHhDYfgasaacH8akY=wiFfYdH8Gipec8Eeeu0xXdbba9frFj0=OqFfea0dXdd9vqai=hGuQ8kuc9pgc9s8qqaq=dirpe0xb9q8qiLsFr0=vr0=vr0dc8meaabaqaciaacaGaaeqabaqabeGadaaakeaacqWGWbaCdaWgaaWcbaGaemysaKKaemitaWKaeGinaqdabeaakiabg2da9maalaaabaGaemOBa42aaSbaaSqaaiabdMeajjabdYeamjabisda0aqabaaakeaacqWGobGtaaGaaCzcaiaaxMaadaqadaqaaiabigdaXaGaayjkaiaawMcaaaaa@3C42@

The subset s is defined as the set of documents that mention IDDM in the abstract. With N_IDDM _as the number of documents in this set we can calculate the expected value (2) of IL4 mentions

*E*[*n*_*IL*4_*IDDM*_] = *N*_IDDM _* *p*_*IL*4 _    (2)

The standard deviation is given by (3)

σ(nIL4_IDDM)=NIDDM*(1−pIL4)*pIL4     (3)
 MathType@MTEF@5@5@+=feaafiart1ev1aaatCvAUfKttLearuWrP9MDH5MBPbIqV92AaeXatLxBI9gBaebbnrfifHhDYfgasaacH8akY=wiFfYdH8Gipec8Eeeu0xXdbba9frFj0=OqFfea0dXdd9vqai=hGuQ8kuc9pgc9s8qqaq=dirpe0xb9q8qiLsFr0=vr0=vr0dc8meaabaqaciaacaGaaeqabaqabeGadaaakeaaiiGacqWFdpWCcqGGOaakcqWGUbGBdaWgaaWcbaGaemysaKKaemitaWKaeGinaqJaei4xa8LaemysaKKaemiraqKaemiraqKaemyta0eabeaakiabcMcaPiabg2da9maakaaabaGaemOta40aaSbaaSqaaiabdMeajjabdseaejabdseaejabd2eanbqabaGccqGGQaGkcqGGOaakcqaIXaqmcqGHsislcqWGWbaCdaWgaaWcbaGaemysaKKaemitaWKaeGinaqdabeaakiabcMcaPiabcQcaQiabdchaWnaaBaaaleaacqWGjbqscqWGmbatcqaI0aanaeqaaaqabaGccaWLjaGaaCzcamaabmaabaGaeG4mamdacaGLOaGaayzkaaaaaa@5416@

The strength of the relationship (4) is then measured by including the real number of occurrences of IL4 in N_IDDM _(n_IL4_IDDM_) in the following equation

cIL4_IDDM=nIL4_IDDM−E[nIL4_IDDM]σ(nIL4_IDDM)     (4)
 MathType@MTEF@5@5@+=feaafiart1ev1aaatCvAUfKttLearuWrP9MDH5MBPbIqV92AaeXatLxBI9gBaebbnrfifHhDYfgasaacH8akY=wiFfYdH8Gipec8Eeeu0xXdbba9frFj0=OqFfea0dXdd9vqai=hGuQ8kuc9pgc9s8qqaq=dirpe0xb9q8qiLsFr0=vr0=vr0dc8meaabaqaciaacaGaaeqabaqabeGadaaakeaacqWGJbWydaWgaaWcbaGaemysaKKaemitaWKaeGinaqJaei4xa8LaemysaKKaemiraqKaemiraqKaemyta0eabeaakiabg2da9maalaaabaGaemOBa42aaSbaaSqaaiabdMeajjabdYeamjabisda0iabc+faFjabdMeajjabdseaejabdseaejabd2eanbqabaGccqGHsislcqWGfbqrcqGGBbWwcqWGUbGBdaWgaaWcbaGaemysaKKaemitaWKaeGinaqJaei4xa8LaemysaKKaemiraqKaemiraqKaemyta0eabeaakiabc2faDbqaaGGaciab=n8aZjabcIcaOiabd6gaUnaaBaaaleaacqWGjbqscqWGmbatcqaI0aancqGGFbWxcqWGjbqscqWGebarcqWGebarcqWGnbqtaeqaaOGaeiykaKcaaiaaxMaacaWLjaWaaeWaaeaacqaI0aanaiaawIcacaGLPaaaaaa@6337@

With N = 2,661,938; n_IL4 _= 20,660; *n*_*IL*4_*IDDM *_= 208 and N_IDDM _= 24,496 we get *c*_*IL*4 _= 1.001.

Coefficients c with high absolute values indicate potentially interesting candidate genes, whereby positive coefficients indicate genes mentioned more frequently than expected (2) and negative coefficients indicate rarely published genes in respect to the expected value. For example, the values for coefficients in multiple sclerosis (MS) range between [-28,9] and for Graves' disease between [-18,15]. The corresponding p-value for a coefficient of 15 as the maximum coefficient for Graves' disease is 3.67 E-51 meaning that the probability to find a coefficient with a higher value converges zero.

### Top 50 genes view

The "Top 50 genes" view is a "hit-list" of genes in the context of all considered autoimmune diseases [26]. This does, however, not imply that these are the most important genes. It merely shows the most published and analysed genes. But again if we apply our ranking coefficient c (4) we get a measure for the strength of the correlation to autoimmune diseases. The coefficient c is calculated in the same way as above except that for the subset s we consider all documents in the AIDB. We have analysed the top 50 genes in the AIDB and calculated precision and recall through random sampling because it was not feasible to evaluate each reference. In such a way we estimated a precision for gene recognition of 88% for the RANK method and 90% for the frequency based method (cf. table 4, column 4). Errors for false recognition include disease names that are also synonyms for genes (IDDM2, IgA-Nephropathy), erroneous linking of cell type names to genes (CD8A), or ambiguous names in the dictionary (ACR, PLF).

### Intersection of gene sets

Intersections of gene sets for different disease concepts are of particular interest. It is widely recognised that persons having one autoimmune disease have an increased susceptibility to other autoimmune disorders. E.g. the MADGC [6] collects information about persons or families that suffer from more than one autoimmune disorder to understand the genes that autoimmune diseases have in common. This has motivated us to provide an interface where intersections of gene sets for different disease concepts can be compiled. For both views, the concept-based view and the MeSH-based view there is the possibility to build these intersections. The intersection interface is located at the bottom of the "Browse by Disease" page and the "Browse by MeSH Term" page respectively [27,28]. A link to the intersection interface is provided at the top of each page to guide the user directly to the interface at the bottom of the page. Two different concepts or MeSH terms can be chosen from a drop-down list. As a result the subset of genes is presented that has co-occurrences with both disease concepts. Searching e.g. the disease term "autoimmune lymphoproliferative" reveals 155 abstracts and 67 genes, searching for "Crohn's disease" reveals 18,137 abstracts and 1,031 genes. Using the intersection mode of the database we could find that almost all genes that co-occur with autoimmune lymphoproliferative (# 57) also occur together with Crohn's disease. Another example is the search for "autoimmune hearing loss". Some of the listed genes are well known in the context of other autoimmune diseases (e.g. HSPA1A and TNFSF5 co-mentioned with over 24 and 70 of the other autoimmune diseases, respectively).

### Evaluation of the database

Given the large number of disease concepts and related genes, a full evaluation of the database is not feasible. However, to provide an evaluation of the system we have selected several diseases which we would like to use for evaluation. We try to answer the following questions: To what extent are we getting the correct documents? To what extent does the co-occurrence approach reveal important links? How does the concept view compare to the MeSH view? A formal evaluation of the gene and protein name recognition performance is not presented here because it was already done in the BioCreAtIvE assessment (see above).

As reference corpus we used the Genetic Association Database (GAD) [29], a manually curated database about genetic associations. All entries in this database have been edited by domain experts. The datasets therefore provide an excellent baseline for evaluating our system.

The evaluation process is as follows: We selected two diseases (multiple sclerosis and Graves' disease) that have a sufficient high number of genes in both databases. Additionally we randomly selected 10 more diseases for evaluation. In the first test we only checked to what extent our database contains the gene-disease associations listed in the GAD. We could show that we retrieved 84% of all gene-disease associations for multiple sclerosis and 100% for Graves' disease (cf. table 5, 1^st ^and 2^nd ^column). For rheumatoid arthritis (RA) and systemic lupus erythematosus (SLE) we yield comparable results (RA: 80%, SLE 82% for the gene disease associations; data not shown). For the other 10 diseases the precision is between 66% and 100% yielding an average of 91% for all 12 considered diseases.

In the second test we checked to what extent we also retrieved the same PubMed references as listed in GAD (table 5, 3^rd ^and 4^th ^column). Here the concept-based and the MeSH-based searches do not differ (data for MeSH not shown). The recall here is 73% for multiple sclerosis and 66% for Graves' disease. The lower values are mainly due to the fact that the HLA genes are not recognised adequately. There is no clear naming convention for HLA genes rendering correct recognition difficult for NER systems (e.g. HLA-DRB1 is often written as DR1). If we exclude the HLA genes the recall will be 77% for multiple sclerosis and 79% for Graves' disease. For the randomly selected diseases the retrieval rate varies between 38% (myasthenia gravis) and 100% (Addison's disease). The bad result for myasthenia gravis is due to the fact that 3 of the 5 genes ("acetylcholine receptor alpha subunit", "AChR" and "AChR beta subunit") are not recognised by ProMiner. The good result for Addison's disease is due to the fact that only 2 genes are in GAD. The average for the retrieval rate taking all 12 diseases into account yields 78%. For detailed results of these tests see Additional file 1 (MS and Graves' disease) and Additional file 2 (for the randomly selected diseases).

The error analysis (cf. table 6) shows that the lower recall is mainly based on the missing recognition of gene and protein names (around 67%) and only to a minor degree (27%) due to missing disease recognition. Lacking dictionary names are one main error source. Sometimes they are excluded from the synonym list due to the high amount of false positive matches (e.g. C6, C7). Another important reason for missing matches are ambiguous gene names. If two or more different gene entries share a synonym which is found in the text, the ProMiner system does only show the match if another synonym from the same gene entry is found in the abstract. As an example we discuss the entry of LRP associated to multiple sclerosis in GAD. The corresponding link to Entrez Gene shows the entry 4035 (LRP1; low density lipoprotein-related protein 1). With the automatic system we did not find this association. The corresponding abstract [30] contains the relevant phrase 'A2M and its receptor low-density lipoprotein receptor-related protein (LRP)'. In Entrez Gene there are 4 gene entries with LPR as synonym (e.g. also 3921: laminin receptor 1; 5786: R-PTP alpha) and more than 10 entries contain the term 'low density lipoprotein-related protein' combined with a number in their name. Specialists can understand which gene is meant because a synonym of LPR1 is 'A2M receptor' but ProMiner and to our knowledge also no other available system is able to resolve this ambiguity. Tests in the BioCreAtIvE assessment (cf. table 3, fly) have shown that accepting also ambiguous matches would lead to higher recall rates (+ 3% for the fly-benchmark) but would decrease precision (-9%) and also F-score (-3%).

To evaluate the quality of the highest ranked genes for the frequency based ranking as well as the RANK-factor based ranking we manually analysed the 50 highest ranked genes for multiple sclerosis in the MeSH setting (1,086 genes). In the left section of table 7 we evaluate the quality of the genes and PubMed references that are in GAD as well as in AIDB (cf. table 7, 1^st ^and 2^nd ^column). For the RANK based method only 3 genes among the top 50 were also in GAD. This is due to the fact that the RANK factor favours associations with few PubMed references. Two of the three PubMed references were correctly retrieved. For the frequency based method 24 genes among the top 50 were also in GAD and 19 of the 24 PubMed references were correctly retrieved (79%). In the right section of table 7 (column 3–6) we evaluate the genes and PubMed references that are **only **in the AIDB. Here the precision of correctly retrieved PubMed references is 81% for the RANK based method and 85% for the frequency based method. Examples for new true associations are listed in column 5 of table 7. CCL5 is a beta-chemokine and has been detected in brain lesions of multiple sclerosis patients. It may serve as a genetic risk marker for MS [31]. Matrix Metalloproteinase 9 (MMP9) is involved in blood-brain barrier (BBB) disruption in active multiple sclerosis [32] whereas glial fibrillary acidic protein (GFAP) may have prognostic value in multiple sclerosis [33]. False associations (cf. table 7, column 6) are mainly based on false recognition of gene names. Here the recognition of CD4 and CD8A as gene names instead of cell types is the most frequent error source. Automatic identification of such false matches is not solved satisfactorily till now and such names could only be recognised due to manual inspection.

In summary it could be shown that the top genes give the researcher a fast overview about relevant genes with acceptable error rates. About half of the genes that are ranked with the frequency based method are also represented in the GAD and further relevant associations could be detected. Using the RANK based method provides further interesting candidate genes with an acceptable precision for PubMed references (81%).

Using MeSH terms does not add any additional false positive errors. We used the PubMed query interface to construct the MeSH term-PMID association table. The situation is different when using UMLS concepts. Here we searched the title and abstract of the Medline entry using concept terms and synonyms as provided by the UMLS. The source of errors lies in the polysemy of the synonyms. Synonyms which are highly ambiguous such as abbreviations like MS, RA, SLE etc. were excluded from the list to limit the number of false positives.

An additional test illustrates the advantage of including disease concepts which are not contained in the MeSH vocabulary. For these diseases we can only provide the associations from the disease concept table. As an example we discussed the results for autoimmune hearing loss and autoimmune hypophysitis. Table 8 shows the results for autoimmune hypophysitis. For this concept the AIDB lists 15 genes with 21 references. Inspecting the references reveals that 17 of the 21 references are correct (81%). The reason for the wrong references is listed in the last column. The results for autoimmune hearing loss are comparable. For this concept 8 genes with 9 references were retrieved and 7 references were correct (77%). These examples show that even in the case of only a few references the AIDB contains candidate genes with an acceptable precision.

## Discussion

Considering the large number of autoimmune diseases and the different medical faculties involved, it is impossible for a single researcher to get an overview of the genes already studied. Suppose a researcher is performing a microarray study for a specific autoimmune disease, i.e. multiple sclerosis. Analysis of differential gene expression reveals a list of several candidate genes. Some of them are known to the researcher and well-discussed in the literature. Others are new in this context. For instance, the official gene name ITGA4 is only mentioned twice in context of multiple sclerosis, but searching for all names listed in the ProMiner dictionary retrieves 52 PubMed abstracts containing different synonyms of ITGA4 (integrin alpha 4, CD49d, VLA-4, alpha 4 subunit of VLA-4 receptor). This is a time- consuming and tedious job where the usage of the AIDB can save a lot of time.

We suppose that the researcher knows the genes for his/her own field of research fairly well, lets say alopecia areata, an autoimmune skin disease. But it is very unlikely that the same researcher, probably a dermatologist, also knows the scientific literature in the area of pancreatitis, which is the subject of gastroenterologists. Following the hypothesis that different autoimmune diseases share similarities at the genomic level, it is a valuable service to provide researchers with candidate genes that were already investigated in another autoimmune disease. A major objective of the AIDB is to serve as an alternative, much more effective way of literature search in the context of autoimmune diseases and genes. It is **not **a database of actual gene-disease associations.

One of the major problems that remains, is the problem of polysemy. Polysemy is the ambiguity of an individual word or phrase that can be used (in different contexts) to express two or more different meanings. Gene or protein names often consist of acronyms of a complicated long form (e.g. ACE = angiotensin converting enzyme, CA2 = carbonic anhydrase 2). ACE does have 158 different long forms beside the enzyme long form which we store in an acronym dictionary. We could ignore the ACE matches in the ProMiner recognition if we find one of these other long forms, otherwise we keep the match. If the long forms of acronyms are not listed in our acronym dictionary this method fails. Furthermore we have no disambiguation strategies if the long form is not available in the text. Another error source is the use of disease names as gene and protein names. Since we found a higher rate of these names in the evaluation for a new dictionary version we have to include strategies for the automatic removal of these names in the next version. Very short names like C6 and C7 as an example for gene names or MS as an example for a disease name are often highly ambiguous and are used in different contexts without using long forms. Here we have two opportunities: Either we can drop every reference without a long form, which will result in a high precision but a lower recall, or we can keep the reference and accept a lower precision for the sake of a higher recall. For names with high frequencies in the Medline corpus we opted for the first possibility to drop all matches. For all other names we accept the possibility of false matches to ensure higher recall. The error analysis showed a lower retrieval rate due to the above mentioned examples which were excluded because of their ambiguous meaning. Furthermore this evaluation shows that such a strategy reaches an average retrieval rate of 78% compared to the entries of the GAD database. Additionally, we found a high rate of new disease-gene associations which occur only in the AIDB. Here a short evaluation of the highest ranking additional matches showed a precision rate of (81–85%) for multiple sclerosis (cf. table 7). With these results we were able to show that the AIDB enables researchers to get an overview of disease-gene associations with a few mouse clicks and reasonable recall and precision rates.

A strong limitation of the database is the missing extraction of the concrete relationships between the diseases and the genes. Currently we do not, for example, distinguish between positive and negative statements. This means that the co-occurrence of CARD15 and psoriasis in "Psoriatic arthritis and CARD15 gene polymorphisms: no evidence for association in the Italian population." (PMID:15140210) adds to the positive number of co-occurrences. The same is true for vague statements according to the pattern "Our results suggest a role of gene x in disease y". Having said this, we emphasise that the primary use case is navigating the autoimmune disease literature.

### Related work

With the ever growing volumes of literature it is becoming more and more essential to apply intelligent methods to extract useful information embedded in literature databases. In the past few years a number of tools and websites have emerged that aim to assist in this task. To the best of our knowledge, there is no tool or database that provides the same information like the AIDB. Here we briefly review some of the related work.

LitMiner [34] is a literature data-mining tool that assists in the identification of gene regulation key players related to a user defined set of key terms within PubMed abstracts. Relationships can be predicted in four categories (genes, chemical compounds, diseases and tissues). The basic underlying method is that of term co-occurrences. Additionally, an overrepresentation score is calculated to filter out the most relevant documents. To allow a curation of the LitMiner predicted relations, the tool is combined with WikiGene, a curation system that applies similar ideas like the Wikipedia Project [35]. Although the scope of LitMiner is much broader in the sense that the diseases are not restricted to autoimmune diseases and that different categories are integrated, the tool is currently not useful in the area of autoimmune diseases. The LitMiner Database currently contains 2,225 diseases, but covers only a fraction of the autoimmune diseases in the AIDB.

CGMIM is a software tool that identifies genetically-associated cancers and candidate genes [36]. This application also uses text-mining and term co-occurrence as a basis for the identification process but the source to be mined is the Online Mendelian Inheritance in Man (OMIM) database. This database has the advantage that it is a manually curated database based on information reported in the scientific literature. It therefore has a particularly high quality. For autoimmune diseases this approach is not feasible because there are only few autoimmune diseases that are mentioned in OMIM.

The Genetic Association Database (GAD) [37] is a publicly available NIH based database of published gene based genetic association studies which contains records of over 5,000 human genetic association studies. This database is curated by experts and therefore has a high quality. The complete content can be downloaded and was used in the evaluation section. In a future version of our database we plan to provide links to the data relevant to autoimmune diseases.

G2D [38] is a database of candidate genes for mapped inherited human diseases. The authors have developed a score system that links genes to diseases based on literature associations of MeSH-D and MeSH-C terms as well as GO functional terms. Due to missing entries for many proteins in the MeSH terms also many gene-disease associations can not be provided with such a method.

Hofmann and Schomburg [39] describe a system where they used co-occurrence of UMLS concepts to assign diseases to enzyme classes in the BRENDA database. The system uses the MetaMap program to identify disease-related concepts by their semantic fields in the UMLS ontology. A support-vector-machine was used to filter out false positives based on their semantic fields. The system yields a precision of 92% and a recall of 50%.

### Future directions

In the current version the construction principles are reflected in the limitation to gene disease associations and in the flat hierarchy of the disease list. There are several directions in which we would like to enhance the AIDB. One way to improve the usability is to introduce a hierarchical structure of disease classes, e.g. Graves' disease is an autoimmune thyroid disease, and allow creative groupings like antibody mediated diseases, T cell mediated diseases, organ specificity, systemic diseases etc. In its next version ProMiner will include protein complex and family names which will expand the coverage to a larger area of names. One other possible expansion is the inclusion of further biomedical entities like drugs, pharmacological substances, environmental factors or polymorphisms.

Approximately one-third of the risk of developing an autoimmune disease can be attributed to hereditary factors; the remainder is thought to be associated with non-inherited conditions. The first version of the autoimmune database concentrates on gene-disease associations simply because the environment-disease link or rather the gene-disease-environment link is poorly understood as of yet. There are hardly any publications about environmental triggers and there are only a handful of known environmental associations. Data in this area is very sparse. Covering all suspected environmental triggers would of course be a very useful and laudable aim, but it would require extensive literature research by domain experts or at least an ontology of environmental triggers, that could be used for literature mining.

For the chemical entity side several very interesting databases were developed in the past few years, e.g. Chemical Entities of Biological Interest (ChEBI) [40] and DrugBank [41,42]. The former lists, as the name suggests, chemical entities that have an influence on biological processes, the latter concentrates on drugs and substance used for drug development. Using this information we could build a co-occurrence matrix similar to the co-occurrence of gene-disease links.

The greatest potential for improvement lies in some kind of ranking of the Medline references. Scanning several hundred references is not practical for the user. It is very desirable to present important or relevant abstracts first. These improvements would require the introduction of text classification methods to analyse the abstracts at a deeper level. Also, the kind of the gene-disease relationship would be very informative. This also requires a deeper analysis of the abstracts. A first step in this direction would be the analysis of the abstracts at the sentence level. The detection of negative associations will be our first focus. Examining the GAD one can see that 38% of the entries for multiple sclerosis are references for negative associations, 34% for positive associations and 28% are not classified. Sorting after positive or negative associations will therefore be a valuable feature. Additional work will also improve the possibilities of searching the database. Here the Gene Ontology [43] can be used to index the database. This would allow users to use Gene Ontology terms to search the database. Finally, it is desirable to optimise the database to gain lower response times.

## Conclusion

To the best of the authors knowledge the AIDB is the first literature-based database that provides gene-disease relationships in the context of all known or suspected autoimmune diseases. In the current version the AIDB can be used as a tool for investigating possible gene-disease associations in the context of autoimmune diseases and navigating the corresponding literature. Although such a dynamically compiled database does not cover all textually available information (around 78% in recall) it provides a high amount of additional information with reasonable precision (around 80%) compared to manually generated content. Due to the high ambiguity of gene names and acronyms used in biomedical context the correct recognition of gene and protein names is the limiting factor for the coverage of the database.

There are three major achievements in this application that allow the comparison of gene sets related to autoimmune diseases on the basis of the published scientific literature:

1) Named entity recognition of gene and protein names and the mapping to unique identifiers done by the ProMiner System.

2) Unifying disease names by using UMLS Concepts and MeSH terms

3) Easy to use user interface that allows to navigate the database content and the original literature

Without a tool of this kind it would be very tedious if not impossible to get an overview of this huge group of diseases.

A database of this kind can be compiled in a semi-dynamic way even with limited resources. The methods and the software used to compile the database can easily be adapted to compile databases for different contexts such as cancer, allergic diseases or inflammatory diseases. However, providing additional biological background knowledge like the classification of the diseases or the genes could greatly enhance the usability of the database.

## Availability and requirements

The Autoimmune Disease Database is freely available for non profit use under the URL 

## Authors' contributions

TK conceived of the study, implemented the database and drafted the manuscript. ÄG participated in the design and helped to draft the manuscript. JF and HTM carried out the gene/protein name recognition and JF is responsible for the ProMiner related evaluation, and helped to draft the manuscript. All authors read and approved the final manuscript.

## Supplementary Material

Additional File 1 Detailed results for MS and Graves' disease. Contains detailed results for the evaluation of the database in comparison to the GAD database for multiple sclerosis and Graves' disease. Click here for file

Additional File 2 Detailed results for randomly selected diseases. Contains detailed results for the evaluation of the database in comparison to the GAD database for 10 randomly selected diseases. Click here for file

## Figures and Tables

**Table 1:** Statistics summarising the content of the Autoimmune Disease Database.

**Parameter**	**Value**	**Description**
N	2,661,938	# abstracts in all of Medline containing proteins/genes recognised by ProMiner
N_Disease_	401,128	# documents that mention at least one autoimmune disease in the title or abstract.
N_DiseaseGene_	85,425	# documents in N_Disease _containing a gene recognised by ProMiner
N_MeSH_	416,742	# documents that have an autoimmune disease as MeSH term
N_MeSHGene_	74,610	# documents in N_MeSH _containing a gene recognised by ProMiner
N_AIDB_	541,690	# documents union of subset N_Disease _and N_MeSH_
N_AIDBGene_	117,021	# documents in N_disease _and N_MeSH _containing a gene recognised by ProMiner
N_g_	132,577	# protein/gene names recognised by ProMiner including synonyms and orthographic variants
N_gdiff_	13,272	# different genes that are recognised by ProMiner in all of Medline
N_gaid_	5,471	# genes in the subset related to autoimmune diseases N_AIDBGene_
MeSH terms	79	# MeSH terms in the context of autoimmune diseases
Concepts	103	# Concepts for autoimmune diseases

Statistics of the AIDB content. Note that the values shown in the table are from 6^th ^of April 2006. The actual values may differ due to an update of the database.

**Table 2:** UMLS concept Takayasu's arteritis.

**Concept**	**Synonym**	**# PMIDs**
Takayasu's Arteritis	Takayasu's Arteritis MeSH	850
	Takayasu's Disease	452
CUI: C0039263	Takayasu Arteritis	387
	PULSELESS DISEASE MeSH	291
	Aortic arch syndrome	246
	TAKAYASU DISEASE	126
	Nonspecific aortoarteritis	97
	Atypical coarctation	73
	Takayasu Syndrome	53
	Middle aortic syndrome	40
	Takayasu's syndrome MeSH	33
	Primary arteritis	22
	Nonspecific arteritis	22
	Takayasu's arteriopathy	12
	Idiopathic aortitis	8
	Martorell syndrome	7
	Aortic arch syndrome	5
	ARTERITIS TAKAYASU	4
	Aortic arch arteritis	3
	Young female arteritis	2
	BRACHIOCEPHALIC ISCHEMIA	2
	TAKAYASUS ARTERITIS	1
	Reverse coarctation	1
	Idiopathic medial aortopathy and arteriopathy	1
	TAKAYASU ARTERIOPATHY	0
	Sclerosing aortitis and arteritis	0
	Occlusive thromboarteriopathy	0
	Raeder-Harbitz syndrome	0
	Reversed coarctation syndrome	0

The concept "Takayasu's arteritis" and the synonyms for this concept as well as their occurrences in Medline. This concept has 29 synonyms and orthographic variants. The terms that are also listed in the MeSH vocabulary are indicated with "MeSH" in column 2. Note, that on the other extreme there are concepts with no synonym like "Psoriasis".

**Table 3:** Performance of the ProMiner system.

**Organism**	**Precision**	**Recall**	**F-score**
Mouse	0.77	0.81	0.79
Yeast	0.97	0.84	0.90
Fly	0.83	0.80	0.82
Fly Accept matches of synonyms associated to up to 3 different Entrez Gene entries	0.74	0.83	0.79
Human	0.86	0.81	0.84

The performance of the ProMiner system for the organisms fly, mouse and yeast was evaluated in the BioCreAtIvE assessment. For the human dictionary we annotated a corpus of 250 abstracts which served as reference corpus to determine recall, precision and F-score. All names are only matched to a gene entry if the recognised synonym is associated only to one gene entry in the corresponding dictionary (called unique matches). Only for the fly organism BioCreAtIvE results with different parameter setting are visualised (c.f. 4^th ^row). Here a recognised name in the text could be matched to up to three different gene entries if the recognised synonym is associated with theses entries.

**Table 4:** Assessment of the "Top 50 genes" page.

	**# Gene-Disease Associations in GAD**	**# correct Gene-Disease Associations not in GAD**	**# False Recognition**	**Precision of Gene Recognition**	**Examples False Recognition**
**RANK based method**	35	9	6	88% (44/50)	**IDDM2 **(insulin dependent diabetes mellitus type II) **CD8A **(CD8-Cells) **IgAN **(IgA-Nephropathy) **ACR **(Albumin-To-Creatinine-Ratio)
**Frequency based method**	34	11	5	90% (45/50)	**IDDM2 **(insulin dependent diabetes mellitus type II) **CD8A **(CD8-Cells) **PLF **(Pulmonary function)

The top 50 genes from the AIDB were evaluated in comparison to GAD (# genes found in GAD, correct genes not found in GAD and falsely recognised genes). The resulting precision of gene recognition is 88% for the RANK based method and 90% for the frequency based method. Erroneously recognised genes are listed in the last column.

**Table 5:** Evaluation of the database content in comparison to the Genetic Association Database (GAD).

**Disease**	**# Gene-disease associations in GAD**	**# Gene-disease associations in AIDB**	**# PubMed references listed in GAD**	**# PubMed references retrieved in AIDB**
multiple sclerosis	57	84% (48/57)	82	73% (60/82)
Graves' disease	14	100% (14/14)	27	66% (18/27)
Addison's Disease	2	100% (2/2)	3	100% (3/3)
sarcoidosis	10	100% (10/10)	13	69% (9/13)
myasthenia gravis	6	66% (4/6)	8	38% (3/8)
alopecia areata	4	75% (3/4)	4	75% (3/4)
Crohn's Disease	14	93% (13/14)	29	93% (27/29)
Psoriasis	16	94% (15/16)	30	73% (22/30)
Ulcerative Colitis	11	90% (10/11)	16	81% (13/16)
Behcet's Disease	8	100% (8/8)	20	85% (17/20)
Narcolepsy	4	100% (4/4)	7	86% (6/7)
Inflamatory bowel Disease	17	94% (16/17)	24	92% (22/24)
**Average**	-	**91%**	-	**78%**

The evaluation results of the AIDB content using gene disease associations referenced in the GAD for the diseases 'multiple sclerosis' and 'Graves' disease' as well as 10 randomly selected diseases are shown. Column 1 lists the number of gene-disease associations extracted out of the GAD database. Column 2 shows the number of these associations found in the AIDB. Column 3 lists the number of PubMed references in GAD and column 4 lists the number of correctly retrieved PubMed references in the AIDB.

**Table 6:** Error analysis for false negative recognition.

	**Gene/Protein Recognition**			**Disease Term Recognition**	**Not found in Abstract**
**Error Class**	Not in dictionary	Ambiguousgene name	Recognition error	Not in dictionary	
**# Errors (%)**	5 (33%)	4 (27%)	1 (6.7%)	4 (27%)	1 (6.7%)
**Error Examples**	C6 C7 Tcr-beta-chain	FAS LRP PLP PD-1		MS	

The error analysis of not recognised associations shows different error classes for missing gene name recognition (columns 1,2,3) and missing disease term recognition (column 4). The first row shows the number of missed references as well as the percentage, the second row shows examples for the different error classes.

**Table 7:** Evaluation of the database content for the top 50 genes in multiple sclerosis.

	**AIDB and GAD**		**Genes and PubMed references only in AIDB**			
**Ranking Method**	**# MS associated Genes**	**# correctly retrieved PubMed references**	**# MS associated Genes**	**# correctly retrieved PubMed references**	**Examples True Recognition**	**Examples False Recognition**

**RANK based Top 50**	3/50	67% (2/3)	47/50	81% (38/47)	MMP27, IFNA16, ADAMTS14	IBD2, Bw3, ABHs, THADA
**Frequency based Top 50**	24/50	79% (19/24)	26/50	85% (22/26)	CCL5, MMP9, GFAP, CCL2, VCAM1, NOS2A	CD4, CD8A, CD86, CD25, CD28, CDR3

Evaluation of the quality of the 50 highest ranked genes in multiple sclerosis for the RANK (first row) and the frequency (second row) based methods. The number of genes that are also in GAD (1^st ^column) and the number of correctly retrieved PubMed references (2^nd ^column) are listed in the left part of the table. The right part (column 3–6) contains the results for the genes that were exclusively found in AIDB. Examples for true recognition are given in column 5 and examples for false recognition are given in column 6.

**Table 8:** Evaluation of the database content for autoimmune hypophysitis.

**Gene Symbol**	**# PMIDs in AIDB**	**Reference correct?**	**PMID**	**Comment**
PRL	4	Y	11683401	
		Y	6325687	
		Y	3923349	
		Y	16392184	
POMC	3	Y	11683401	
		Y	2840382	
		Y	1310997	
GNRH1	2	Y	3923349	
		Y	2840382	
CD4	1	N	15493593	CD4 T cell recognised
CYP19A1	1	Y	15493593	
COL14A1	1	N	16425001	"und" recognised as synonym
INA	1	Y	15234547	
LY75	1	Y	15493593	
CTLA4	1	Y	16224277	
PTPRC	1	Y	15493593	
TG	1	Y	1310997	
AGMX2	1	N	15963060	"with growth hormone deficiency" as synonym
TNFRSF11B	1	Y	15493593	
TNFRSF25	1	N	7800142	DR3 recognised as synonym
TPO	1	Y	1310997	

Results for autoimmune hypophysitis. The term "autoimmune hypophysitis" is not in the MeSH vocabulary. 81% of the documents were correctly retrieved.
