# Supplementary material for: The Autoimmune Disease Database: a dynamically compiled literature-derived database
Source: BMC Bioinformatics. 2006 Jun 27;7:325. doi: 10.1186/1471-2105-7-325 (PMC1525205; doi:10.1186/1471-2105-7-325)
Supplement: Additional File 1 — Detailed results for MS and Graves' disease. Contains detailed results for the evaluation of the database in comparison to the GAD database for multiple sclerosis and Graves' disease. [file 1471-2105-7-325-S1.doc]

### Additional file 1 – Detailed results for multiple sclerosis and Graves’ disease

| Multiple Sclerosis | | | | | |
| --- | --- | --- | --- | --- | --- |
| **Gene Symbol** | **# of PMIDs in AIDB** | **Gene listed in AIDB[Y/N]** | **Reference listed in AIDB[Y/N]** | **PMID** | **Comment** |
| A2M | 12 | Y | N | 11036822 | alpha2-macroglobulin not recognized synonym |
|  |  |  | Y | 11498265 |  |
| ADRB2 | 2 | Y | Y | 12474986 |  |
| APOC2 | 2 | Y | N | 10335523 | apolipoprotein C-II not recognized |
| APOE | 54 | Y | N | 11171894 | MS not in disease list |
|  |  |  | Y | 11836653 |  |
|  |  |  | Y | 10406990 |  |
|  |  |  | Y | 10888365 |  |
|  |  |  | Y | 11552016 |  |
|  |  |  | Y | 12926843 |  |
| C6 | 0 | N | N | 10496188 | Not recognized by ProMiner |
| C7 | 0 | N | N | 10496188 | Not recognized by ProMiner |
| CCR5 | 11 | Y | N | 12451219 | MS not in disease list |
| CD24 | 2 | Y | Y | 14657362 |  |
| CD28 | 50 | Y | Y | 12864988 |  |
| CTLA4 | 55 | Y | Y | 12458055 |  |
|  |  |  | Y | 10082437 |  |
|  |  |  | Y | 12507781 |  |
|  |  |  | Y | 10408973 |  |
|  |  |  | Y | 11239948 |  |
|  |  |  | Y | 12864988 |  |
|  |  |  | Y | 14975605 |  |
| ESR1 | 13 | Y | Y | 12098649 |  |
| FAS | 0 | N | N | 9553736 | Not recognized by ProMiner |
| FASLG | 0 | N | N | 11438180 | Not recognized by ProMiner |
| GABRA3 | 1 | Y | Y | 9561979 |  |
| HLA |  | Y | N | 7911477 | HLA DR2 used instead of HLA-DRB2 |
|  |  |  | Y | 10468508 |  |
|  |  |  | N | 12120698 | HLA DQAI used instead of HLA DQA1 |
|  |  |  | Y | 9258248 |  |
|  |  |  | N | 3492235 |  |
|  |  |  | N | 9756407 | Generell reference to HLA-DP |
| HLA-DRB1 |  | N | N | 14669136 | ProMiner recoginzes HLA-DRB4 |
| ICAM1 | 141 | Y | Y | 11081805 |  |
| IFN alpha/beta | 131 | Y | Y | 10087402 |  |
| IFNAR1 | 1 | Y | Y | 12618863 |  |
| IFNG | 752 | Y | Y | 9818947 |  |
|  |  |  | Y | 10505747 |  |
| IL10 | 324 | Y | Y | 12101075 |  |
| IL1B | 126 | Y | N | 10025794 | MS not in disease list |
| IL1RN | 22 | Y | N | 10025794 | MS not in disease list |
|  |  |  | N | 8765338 | interleukin 1-receptor-antagonist |
| IL2 | 268 | Y | Y | 12409183 |  |
| IL4 | 283 | Y | Y | 9184650 |  |
| IL6 | 193 | Y | Y | 11072134 |  |
|  |  |  | Y | 11196678 |  |
|  |  |  | Y | 11072134 |  |
| IRF1 | 11 | Y | Y | 11196707 |  |
| LRP |  | N | N | 11498265 | LRP not recognized by ProMiner |
| MBP | 1173 | Y | Y | 9460711 |  |
|  |  |  | Y | 7523603 |  |
|  |  |  | Y | 1691612 |  |
|  |  |  | Y | 7685461 |  |
|  |  |  | Y | 9482678 |  |
| MEFV | 4 | Y | Y | 12700594 |  |
| MOG | 260 | Y | Y | 9436746 |  |
|  |  |  | Y | 7593547 |  |
|  |  |  | Y | 9493637 |  |
| MPO | 15 | Y | Y | 10742562 |  |
| NOS1 |  | Y | Y | 14759629 |  |
| NOTCH3 |  | Y | Y | 11413271 |  |
| PDCD1 |  | N | N | 15912506 | PD-1 not recognized by ProMiner |
| PECAM1 | 10 | Y | Y | 10713357 |  |
| PLP1 | 64 | Y | N | 9460711 | PLP not recognized by ProMiner |
| PNMT | 1 | Y | Y | 11958827 |  |
| PRKCA | 1 | Y | - | - | Only OMIM reference in GAD |
| PTPN22 | 3 | Y | Y | 15934099 |  |
| PTPRC | 40 | Y | Y | 12810785 |  |
|  |  |  | Y | 11101853 |  |
| SH2D2A | 3 | Y | Y | 11528519 |  |
| SLC11A1 | 4 | Y | Y | 11358358 |  |
| SPP1 | 9 | Y | N | 12928913 |  |
| T cell receptor betachain | 39 | Y | N | 1672869 | Gene synonym not in database |
| TAP 2 transporter | 1 | Y | Y | 7929801 |  |
| TAP1 | 7 | Y | Y | 7929801 |  |
| Tcell receptor alpha | 20 | Y | Y | 8857743 |  |
| TGF | 160 | Y | Y | 10335519 |  |
| TNF | 764 | Y | Y | 10522904 |  |
|  |  |  | Y | 10773851 |  |
| TNFRSF1A | 46 | Y | Y | 11598334 |  |
| VDR | 2 | Y | Y | 10465499 |  |
|  |  |  | N | 10967184 | Not recognized by ProMiner  VDRG or vitamin D receptor gene |
| vitamin D-binding protein | 3 | Y | Y | 12044990 |  |

| Graves’ Disease | | | | | |
| --- | --- | --- | --- | --- | --- |
| **Gene Symbol** | **# of concept PMIDs in AIDB** | **Gene listed in AIDB[Y/N]** | **Reference listed in AIDB[Y/N]** | **PMID** | **Comment** |
| C4A | 7 | Y | Y | 2570594 |  |
| CTLA4 | 75 | Y | Y | 12724780 |  |
|  |  |  | Y | 7829637 |  |
|  |  |  | Y | 9231050 |  |
| GC | 1 | Y | Y | 12050214 |  |
| HLA |  | Y | N | 6896102 | HLA-DR3 |
|  |  |  | N | 8157715 |  |
|  |  |  | Y | 11272094 | HLA-DQA1 |
|  |  |  | Y | 8306482 | HLA-DQA1 |
|  |  |  | N | 6932402 |  |
|  |  |  | N | 2567295 |  |
|  |  |  | Y | 10770195 | HLA-DRB3 |
| HLADQA1 *0501 | 5 | Y | N | 8501164 | HLA-DRB3 |
| ICAM1 | 72 | Y | Y | 14557478 |  |
| IL13 | 7 | Y | N | 10719301 | Graves Disease not mentioned in abstract |
|  |  |  | Y | 14510917 |  |
| IL1A | 24 | Y | Y | 8954062 |  |
| IL1RN | 5 | Y | Y | 9678537 |  |
|  |  |  | Y | 7530255 |  |
|  |  |  | N | 8954062 | „Interleukin-1 receptor“ not in DB |
| IL4 | 54 | Y | Y | 11502824 |  |
| INS | 21 | Y | N | 10211611 |  |
| LMP2 | 5 | Y | Y | 10468973 |  |
| THRB | 5 | Y | Y | 8748133 |  |
| TSH- R | 511 | Y | Y | 9777748 |  |
|  |  |  | Y | 7883818 |  |
|  |  |  | Y | 9086566 |  |
