# Supplementary material for: The Autoimmune Disease Database: a dynamically compiled literature-derived database
Source: BMC Bioinformatics. 2006 Jun 27;7:325. doi: 10.1186/1471-2105-7-325 (PMC1525205; doi:10.1186/1471-2105-7-325)
Supplement: Additional File 2 — Detailed results for randomly selected diseases. Contains detailed results for the evaluation of the database in comparison to the GAD database for 10 randomly selected diseases. [file 1471-2105-7-325-S2.doc]

### Additional file 2 – Detailed results for the 10 randomly selected diseases

| Inflammatory Bowel Disease | | | | | |
| --- | --- | --- | --- | --- | --- |
| **Gene Symbol** | **# of PMIDs in AIDB** | **Gene listed in AIDB[Y/N]** | **Reference listed in AIDB[Y/N]** | **PMID** | Comment |
| beta7 integrin | 5 | Y | Y | 11781713 |  |
| CARD15 | 330 | Y | Y | 15052696 |  |
|  |  |  | Y | 11425413 |  |
|  |  |  | Y | 11910337 |  |
|  |  |  | Y | 13680363 |  |
|  |  |  | Y | 15046221 |  |
| CCR5 |  | Y | Y | 11354628 |  |
| CD11 | 20 | Y | Y | 11862384 |  |
| CD14 | - | N | N | 12126249 |  |
| F2 | 56 | Y | Y | 11383586 |  |
| HLA-DP | 1 | Y | Y | 12073072 |  |
| ICAM1 | 111 | Y | Y | 11726228 |  |
|  |  |  | Y | 12477764 |  |
| IFNGR1 | 1 | Y | Y | 10207725 |  |
| IL1B | 232 | Y | Y | 9568467 |  |
| IL1RN | 83 | Y | Y | 9568467 |  |
| IL4R | 6 | Y | Y | 10663555 |  |
| MLH1 |  | Y | Y | 12011151 |  |
| NFkB | 140 | Y | N | 12019209 | NF(-kappa)B not recognized |
| SLC11A1 | 4 | Y | Y | 11929588 |  |
|  |  |  | Y | 10207725 |  |
| TNF | 790 | Y | Y | 12811429 |  |
|  |  |  | Y | 12019209 |  |
| TPMT | 72 | Y | Y | 12940924 |  |

| Myasthenia Gravis | | | | | |
| --- | --- | --- | --- | --- | --- |
| **Gene Symbol** | **# PMIDs in AIDB** | **Gene listed in AIDB[Y/N]** | **Reference listed in AIDB[Y/N]** | **PMID** | Comment |
| AChR | - | N | N | 14981744 | Gene not recognized by ProMiner |
| acetylcholine receptor alphasubunit | - | N | N | 8738961 | Gene not recognized by ProMiner |
| AChR betasubunit | - | N | N | 9307231 | Gene not recognized by ProMiner |
| CTLA4 | 20 | Y | Y | 9688341 |  |
| HLA | - | Y | N | 8370765 | DRB1 not associated with HLA-DRB1 |
|  |  |  | N | 9817446 | HLA-B8 not recognized |
| IL1 | 2 | Y | Y | 11777547 |  |
| TNF | 41 | Y | Y | 9688335 |  |

| Narcolepsy | | | | | |
| --- | --- | --- | --- | --- | --- |
| **Gene Symbol** | **# of PMIDs in AIDB** | **Gene listed in AIDB[Y/N]** | **Reference listed in AIDB[Y/N]** | **PMID** | Comment |
| HCRT | 163 | Y | Y | 11148249 |  |
| HLA | 22 | Y | Y | 10777671 |  |
|  |  |  | N | 1973684 | Allele not recognized by ProMiner |
|  |  |  | Y | 11382879 |  |
| TNF | 12 | Y | Y | 12601524 |  |
|  |  |  | Y | 10488740 |  |
| TNFR2 | 3 | Y | Y | 11144293 |  |

| Behcet’s Disease | | | | | |
| --- | --- | --- | --- | --- | --- |
| **Gene Symbol** | **# of PMIDs in AIDB** | **Gene listed in AIDB[Y/N]** | **Reference listed in AIDB[Y/N]** | **PMID** | Comment |
| ACE | 4 | Y | N | 14672905 | Not recognized by ProMiner |
| HLA | 66 | Y | Y | 10205273 |  |
|  |  |  | Y | 11508592 |  |
|  |  |  | Y | 8213969 |  |
|  |  |  | Y | 11796403 |  |
|  |  |  | Y | 10440244 |  |
|  |  |  | Y | 1359669 |  |
|  |  |  | Y | 12068141 |  |
|  |  |  | Y | 12632436 |  |
|  |  |  | Y | 9714478 |  |
|  |  |  | Y | 10513813 |  |
| F2 | 18 | Y | Y | 11820731 |  |
|  |  |  | Y | 15077257 |  |
| F5 | 18 | Y | Y | 15077257 |  |
| IL1A | 8 | Y | Y | 12730545 |  |
| MICA | 13 | Y | N | 10519365 | Not recognized by ProMiner |
|  |  |  | N | 9037047 | Not recognized by ProMiner |
| NOS3 | 2 | Y | Y | 14583572 |  |
|  |  |  | Y | 11908569 |  |
| platelet glycoprotein Ia | 1 | Y | Y | 12412731 | Official name ITGA2 |

| Sarcoidosis | | | | | |
| --- | --- | --- | --- | --- | --- |
| **Gene Symbol** | **# PMIDs in AIDB** | **Gene listed in AIDB[Y/N]** | **Reference listed in AIDB[Y/N]** | **PMID** | Comment |
| ACE | 376 | Y | Y | 10362030 |  |
|  |  |  | Y | 10672133 |  |
|  |  |  | Y | 9713444 |  |
|  |  |  | N | 9713438 |  |
| CARD15 | 17 | Y | Y | 14597055 |  |
| CFTR | 3 | Y | Y | 10980579 |  |
| HLA-B | 4 | Y | N | 14656748 |  |
| HLA-DRB1 | 34 | Y | Y | 14508706 |  |
| IL1B | 41 | Y | Y | 11127488 |  |
| macrophage migration inhibitory factor | 3 | Y | N | 12180727 |  |
| SCGB1A1 | 3 | Y | N | 14551164 | Clara cell 10-kD protein |
| LMP7 | 3 | Y | Y | 9458120 |  |
| NRAMP | 3 | Y | Y | 10837363 |  |

| Ulcerative Colitis | | | | | |
| --- | --- | --- | --- | --- | --- |
| **Gene Symbol** | **# PMIDs in AIDB** | **Gene listed in AIDB[Y/N]** | **Reference listed in AIDB[Y/N]** | **PMID** | Comment |
| ABCB1 | 13 | Y | Y | 12512026 |  |
|  |  |  | Y | 14755848 |  |
| HLA | 19 | Y | Y | 11696218 |  |
| IKBL | 1 | Y | Y | 11113070 |  |
| IL11 | 5 | Y | Y | 12486609 |  |
| IL1RN | 53 | Y | Y | 8119534 |  |
|  |  |  | Y | 1124788811592376 |  |
|  |  |  | Y | 92039419373766 |  |
|  |  |  | Y | 105000629858044 |  |
| KRAS | 8 | Y | N | 8160776 |  |
| MLH1 | 8 | Y | Y | 9230812 |  |
| MUC3A | - | N | N | 9931338 |  |
| TCRA | 6 | Y | N | 2571544 |  |
| TNF | 243 | Y | Y | 11904678 |  |
| TP53 | 87 | Y | Y | 11737306 |  |
|  |  |  | Y | 8160776 |  |

| Crohn’s Disease | | | | | |
| --- | --- | --- | --- | --- | --- |
| **Gene Symbol** | **# of PMIDs in AIDB** | **Gene listed in AIDB[Y/N]** | **Reference listed in AIDB[Y/N]** | **PMID** | Comment |
| CARD15 | 274 | Y | Y | 12360101 |  |
|  |  |  | Y | 12020527 |  |
|  |  |  | Y | 11926563 |  |
|  |  |  | Y | 12508397 |  |
|  |  |  | Y | 12210321 |  |
|  |  |  | Y | 12631669 |  |
|  |  |  | Y | 14638352 |  |
|  |  |  | Y | 12876263 |  |
|  |  |  | Y | 15046222 |  |
|  |  |  | Y | 14765395 |  |
|  |  |  | Y | 15002819 |  |
|  |  |  | Y | 15008984 |  |
|  |  |  | Y | 15056084 |  |
|  |  |  | Y | 12940436 |  |
|  |  |  | Y | 15024686 |  |
| CD14 | - | N | N | 11843056 |  |
|  |  |  | N | 12940436 |  |
| CD19 | 7 | Y | Y | 12215898 |  |
| HLA | 32 | Y | Y | 8732477 |  |
| IL16 | 4 | Y | Y | 12706406 |  |
| IL4 | 64 | Y | Y | 11294568 |  |
| IL4R | 3 | Y | Y | 11294568 |  |
| NOD2 | -* | Y | Y | 11385576 | * Synonym for CARD15 |
| NRAMP | 2 | Y | Y | 9288120 |  |
| SLC22A4 | 14 | Y | Y | 15107849 |  |
| SLC22A5 | 10 | Y | Y | 15107849 |  |
| TNF | 461 | Y | Y | 11196680 |  |
|  |  |  |  | 12190096 |  |
| TNFRSF1B | 48 | Y | Y | 11196680 |  |
| VDR | 3 | Y | Y | 10896912 |  |

| Alopecia Areata | | | | | |
| --- | --- | --- | --- | --- | --- |
| **Gene Symbol** | **# of PMIDs in AIDB** | **Gene listed in AIDB[Y/N]** | **Reference listed in AIDB[Y/N]** | **PMID** | Comment |
| HLA | - | N | N | 12589427 | Generell reference to HLA class II alleles |
| IL1A | 8 | Y | Y | 11703512 |  |
| IL1B | 13 | Y | Y | 11703512 |  |
| IL1RN | 8 | Y | Y | 8077705 |  |

| Psoriasis | | | | | |
| --- | --- | --- | --- | --- | --- |
| **Gene Symbol** | **# of PMIDs in AIDB** | **Gene listed in AIDB[Y/N]** | **Reference listed in AIDB[Y/N]** | **PMID** | Comment |
| Alpha 1 antitrypsin | 24 | Y | Y | 6155027 |  |
| CDSN | 30 | Y | Y | 12472658 |  |
|  |  |  | Y | 10332047 |  |
| HLA | - | Y | N | 11194889 | HLA-C not recognized |
|  |  |  | N | 10888604 | HLA-C not recognized |
|  |  |  | N | 6984837 |  |
|  |  |  | N | 11069619 | HLA-C not recognized |
|  |  |  | Y | 11556968 |  |
|  |  |  | Y | 10545595 |  |
|  |  |  | Y | 10599883 |  |
|  |  |  | N | 8504697 |  |
|  |  |  | N | 12648225 |  |
| IL10 | 86 | Y | Y | 10469306 |  |
| IL12B | 6 | Y | Y | 12413772 |  |
| IL1RN | 25 | Y | Y | 9039327 |  |
| IRF2 | 5 | Y | Y | 14962090 |  |
| MICA | 4 | Y | Y | 10691930 |  |
|  |  |  | N | 10323458 | MICA-A9 not recognized |
| MMP2 | 7 | Y | Y | 12077518 |  |
| PTPN22 | 2 | Y | Y | 15934099 |  |
| S100A2 | 2 | Y | Y | 11260185 |  |
| SKALP | 28 | Y | Y | 9727750 |  |
| SLC9A3R1 | 3 | Y | Y | 14608357 |  |
| TAP | - | N | N | 12648225 |  |
| TNF | 329 | Y | Y | 10469306 |  |
|  |  |  | Y | 9395887 |  |
|  |  |  | Y | 9326391 |  |
|  |  |  | Y | 11851889 |  |
| VDR | 28 | Y | Y | 9886274 |  |
|  |  |  | Y | 12071154 |  |

| Addison’s Disease | | | | | |
| --- | --- | --- | --- | --- | --- |
| **Gene Symbol** | **# of PMIDs in AIDB** | **Gene listed in AIDB[Y/N]** | **Reference listed in AIDB[Y/N]** | **PMID** | Comment |
| CTLA4 | 8 | Y | Y | 10197076 |  |
|  |  |  | Y | 10690877 |  |
| VDR | 1 | Y | Y | 12444895 |  |
